# Propofol Inhibits Proliferation and Augments the Anti-Tumor Effect of Doxorubicin and Paclitaxel Partly Through Promoting Ferroptosis in Triple-Negative Breast Cancer Cells

**DOI:** 10.3389/fonc.2022.837974

**Published:** 2022-03-28

**Authors:** Chen Sun, Pan Liu, Lijian Pei, Mengyun Zhao, Yuguang Huang

**Affiliations:** ^1^ Department of Anesthesiology, Peking Union Medical College Hospital, Peking Union Medical College, Chinese Academy of Medical Sciences, Beijing, China; ^2^ Joint Laboratory of Anesthesia and Pain, Peking Union Medical College, Chinese Academy of Medical Sciences, Beijing, China; ^3^ Department of Human Anatomy, Histology and Embryology, Institute of Basic Medical Sciences, Peking Union Medical College, Chinese Academy of Medical Sciences, Beijing, China; ^4^ Department of Hematology, Zhongnan Hospital of Wuhan University, Wuhan, China; ^5^ Outcomes Research Consortium, Cleveland, OH, United States

**Keywords:** propofol, triple-negative breast cancer, ferroptosis, propofol injectable emulsion, fospropofol disodium

## Abstract

**Background:**

Triple-negative breast cancer (TNBC) is relatively common in women and is associated with a poor prognosis after surgery and adjuvant chemotherapy. Currently, the mechanism underlying the relationship between propofol and breast cancer is controversial and limited to cell apoptosis. Moreover, there are only a few studies on the effect of propofol on the chemotherapeutic sensitivity of TNBC cells. Therefore, this study explored whether propofol and its commonly used clinical formulations affect the proliferation and chemotherapeutic effects on TNBC cells by regulating cell ferroptosis.

**Methods:**

We selected MDA-MB-231 cells, and the effects of propofol, propofol injectable emulsion (PIE), or fospropofol disodium, alone or combined with doxorubicin or paclitaxel on cell viability, apoptosis, intracellular reactive oxygen species (ROS) accumulation, ferroptosis-related morphological changes, intracellular Fe^2+^ levels, and the expression and localization of ferroptosis-related proteins were investigated.

**Results:**

We found that propofol significantly inhibited MDA-MB-231 cell proliferation, and all three propofol formulations augmented the anti-tumor effects of doxorubicin and paclitaxel. The results from the ROS assay, transmission electron microscopy, intracellular Fe^2+^ assay, western blotting, and multiplex immunohistochemistry revealed that propofol not only induced apoptosis but also triggered ferroptosis-related changes, including morphological changes of mitochondria, increased intracellular ROS levels, and intracellular iron accumulation in MDA-MB-231 cells. The ferroptosis-related p53-SLC7A11-GPX4 pathway was also altered under different treatment propofol, doxorubicin, or paclitaxel regimens.

**Conclusion:**

Propofol showed anti-proliferation effects on TNBC cells and could be a potential adjuvant to enhance the chemotherapeutic sensitivity of TNBC cells partly by promoting cell ferroptosis.

## Introduction

Breast cancer is the most common cancer in women and the second leading cause of cancer death (1, 2). Among breast cancers, triple-negative breast cancer (TNBC), which lacks the expression of hormone receptors and human epidermal growth factor receptor 2 (HER2), is associated with a higher rate of recurrence and poorer prognosis after surgery and adjuvant chemotherapy (3). Therefore, it is necessary to optimize treatment strategies for TNBC.

Surgery is currently the primary treatment for breast cancer. As one of the most commonly used intravenous anaesthetics (4), propofol (2,6-diisopropylphenol) has also shown many non-anesthetic effects, such as inhibitory effect on tumor in recent investigations (5). Most studies have found that propofol inhibits proliferation, migration, and invasion of cancer cells and promotes cell apoptosis *in vitro* by regulating the expression of various signaling pathways and non-coding microRNAs (6, 7), suppressing tumor growth and metastasis *in vivo* (8, 9), and increasing sensitivity to chemotherapeutics (10, 11).

The current findings remain controversial in terms of breast cancer. In clinical research, some studies demonstrated that compared with inhalation anesthetics, using propofol during surgery could improve the prognosis of patients with breast cancer (12); however, this effect has not been observed in other studies (13). As for *in vitro* studies, most studies showed that propofol inhibited the proliferation of breast cancer cells through different pathways. For example, propofol induces MDA-MB-435 cell apoptosis by downregulating the miR-24 signaling pathway (14). In addition, by suppressing miR-21 expression, propofol could inhibit the proliferation and epithelial-mesenchymal transition of MCF-7 cells (15). However, researchers also found that propofol may promote the proliferation and metastasis of breast cancer (16, 17). Besides the controversy, the proposed underlying mechanisms in the aforementioned *in vitro* studies are mainly based on cell apoptosis and related pathways, and they only choose propofol itself in their experiments, without including the commonly used clinical propofol formulations, such as propofol injectable emulsion (PIE), limiting the clinical value. Moreover, there are few studies on the effect of propofol on the chemotherapeutic sensitivity of TNBC cells.

Ferroptosis is an iron-dependent form of regulated cell death driven by excessive lipid peroxidation, which is morphologically, biochemically, and genetically distinct from apoptosis, necrosis, and autophagy (18). Furthermore, it is reported to be involved in various diseases such as cancer, stroke, and diabetes (19–22). Ferroptosis is characterized by the accumulation of intracellular iron and lipid reactive oxygen species (ROS) and is regulated by several pathways related to intracellular redox reactions such as the SLC7A11-GPX4 (23) and the ubiquinone-FSP1-ubiquinol axes (24, 25). In the past decade, ferroptosis has been implicated in the development and therapeutic response of various tumor types. Previous studies have suggested that inducing ferroptosis may be an effective strategy for tumor treatment and preventing acquired resistance to multiple chemotherapeutic agents (19).

In this study, we aimed to explore whether propofol, PIE, and fospropofol disodium affect the proliferation of TNBC cells and chemotherapeutic effects by regulating cell ferroptosis, hoping to provide novel treatment strategies for patients with TNBC.

## Materials and Methods

### Cell Lines and Reagents

The human breast cancer cell line MDA-MB-231, a TNBC cell line, was obtained from China Infrastructure of Cell Line Resource (Beijing, China). Propofol was obtained from Sigma-Aldrich (1572503; St. Louis, MO, USA), with PIE from AstraZeneca (Cambridge, UK) and fospropofol disodium from Yichang Humanwell Pharmaceutical Co., Ltd. (LB52200301; Hubei, China). Doxorubicin (A1832) and paclitaxel (A4393) were purchased from Applied Biosystems (Houston, TX, USA).

### Cell Culture and Drug Application

MDA-MB-231 cells were cultured in Roswell Park Memorial Institute 1640 medium (C11875500BT; Gibco, Grand Island, NY, USA) containing 10% fetal bovine serum (FBS) (AQmv09900; Analysis Quiz, Uruguay, South America) and 1% penicillin-streptomycin (15140-122; Gibco) in an incubator with 5% CO_2_ at 37°C. Propofol and fospropofol disodium were first dissolved in dimethyl sulfoxide (DMSO) (D2650; Sigma Aldrich) and normal saline respectively to prepare 50mg/ml store solution, and then diluted into 2, 5, 10 and 20 µg/ml using complete cell culture medium. PIE, 10mg/ml originally, was directly diluted in complete medium at 2, 5, 10, and 20 µg/ml. Doxorubicin and paclitaxel were also dissolved in DMSO and then in complete medium at 0.01, 0.1, 0.5, 1, 5, and 10 µg/ml. The final concentration of DMSO was less than 0.1%. All drugs were administered at different concentrations when cell growth reached 60-70% confluence, and treatment continued for 24 h.

### Cell Viability Assay

The viability of MDA-MB-231 cells was determined using a Cell Counting Kit-8 (CCK-8, CK04; Dojindo Laboratories, Japan). Briefly, the cells were digested with 0.05% trypsin-EDTA (25300-062; Gibco) and then inoculated into Costar^®^ 96-well plates (Corning Inc., Corning, NY, USA) at a cell density of 2 × 10^4^ cells per well. When the fusion rate of cells increased to 60-70% after overnight culture, the cells were replenished with fresh medium containing drugs at the indicated concentrations. The cells were then incubated for 24 h and subsequently replenished with fresh medium containing 10 µl of CCK-8 reagent for each well. After fully reacting in a 37°C incubator in the dark for another 2 h, an Epoch Microplate Reader (BioTek, Winooski, VT, USA) was used to measure the optical density (OD) of the cells in each well at 450 nm. The percentage of viable cells was determined using the following formula: cell viability (%) = (treatment group OD – blank group OD)/(control group OD – blank group OD) × 100%. Each experiment was conducted in triplicate.

### Flow Cytometry

An Annexin V-FITC Apoptosis Detection Kit (AD10; Dojindo Laboratories, Kumamoto, Japan) was used to analyze MDA-MB-231 cell apoptosis. Briefly, the cells were seeded into Costar^®^ 6-well plates (Corning Inc.) and digested with 0.05% trypsin after treatment with the drugs for 24 h, then collected and washed twice with ice-cold phosphate-buffered saline (PBS, 1×; Hyclone Laboratories Inc., Logan, UT, USA). Thereafter, a 1× Annexin V binding solution was added to make a cell suspension at a final concentration of 1 × 10^6^ cells/ml. The cells were then stained with Annexin V-FITC and propidium iodide (PI) for 15 min in the dark at room temperature (RT). After adding 400 µl of 1× Annexin V binding solution into each tube of the cell suspension, the cells were loaded onto a flow cytometer (Accuri C6 Plus; BD BioSciences, Franklin Lakes, NJ, USA) within 1 h. The results of three independent experiments were analyzed using BD Accuri C6 Plus software, according to the manufacturer’s instructions.

### Intracellular Reactive Oxygen Species (ROS) Assay

The DCFDA/H2DCFDA-Cellular ROS Assay Kit (ab113851; Abcam, Cambridge, UK) was used to analyze intracellular ROS levels in MDA-MB-231 cells. Briefly, the cells were grown in eight-chambered slides (155411; Nalge Nunc International Corporation, Rochester, NY, USA) to an appropriate density before treatment with the drugs. After 24 h of cultivation in 5% CO_2_ at 37°C, cells were washed twice with 1× buffer and stained with diluted DCFDA solution for 45 min at 37°C in the dark. Subsequently, the cells were washed twice with 1× buffer and viewed using a real-time live-cell laser scanning confocal microscope (UltraVIEW VOX; PerkinElmer, Waltham, MA, USA) with a filter set appropriate for fluorescein (FITC) under low light conditions. Intracellular ROS-positive cells were counted, and their ratio was calculated by comparing them with total cells in each microscope field.

### Transmission Electron Microscopy (TEM)

MDA-MB-231 cells were trypsinized and centrifuged at 1 000 rpm for 5 min, followed by fixation in 2.5% glutaraldehyde (EM Grade, P1126; Solarbio Life Sciences, Beijing, China) at 4°C overnight. Next, the cell samples were fixed in 1% osmium acid, dehydrated, and embedded in molds in a standard fashion. Afterwards, appropriate areas were selected, and ultrathin sections (0.08 µm) were stained with lead citrate and uranyl acetate for 5-10 min at approximately 95°C. Finally, the sections were analyzed by TEM (JEM-1400Plus; JEOL Ltd., Tokyo, Japan).

### FerroOrange Iron Assay

MDA-MB-231 cells were inoculated into eight-chambered slides and cultivated until the fusion rate reached 60-70%. Next, the original medium was discarded and replaced with fresh medium containing the drugs at the indicated concentrations. After incubation at 37°C for 24 h, the cells were washed with serum-free medium thrice. Subsequently, 1 µmol/L FerroOrange working solution (F374; Dojindo Laboratories Inc.) was added into each chamber and cultivated in 5% CO_2_ at 37°C for 30 min. Thereafter, cell samples were imaged using a real-time live-cell laser scanning confocal microscope (UltraVIEW VOX; PerkinElmer, Inc.). The relative mean fluorescence intensity (MFI) of intracellular ferrous ions in different microscopic fields was calculated and analyzed using ImageJ software.

### Western Blotting

MDA-MB-231 cells were lysed in RIPA buffer (R0020; Solarbio Life Sciences) for 30 min at 4°C after treatment with the drugs for 24 h. The lysates were centrifuged at 12 000 rpm for 15 min at 4°C, and the supernatant containing total protein was harvested and then denatured. Next, protein concentrations were determined using a bicinchoninic acid (BCA) assay system (Beyotime, Shanghai, China). Protein samples were then separated by 10 or 15% sodium dodecyl sulfate-polyacrylamide (SDS-PAGE) gel electrophoresis, according to molecular weight, and then electrophoretically transferred to polyvinylidene fluoride (PVDF) membranes (with 0.45-µm diameter pores). After 2 h of blocking with 5% skim milk at RT, the PVDF membranes were incubated in primary antibodies diluted in universal antibody diluent (WB500D; NCM Biotech, Suzhou, China) at 4°C overnight; the primary antibodies included anti-caspase-3 (1:5 000; Abcam, ab32351), anti-Bcl-2 (1:1 000; Abcam, ab182858), anti-GPX4 (1:2 000; Abcam, ab125066), anti-SLC7A11 (1:2 000; Abcam, ab175186), anti-p53 (1:1 000; CST, #2524), anti-ubiquinol-cytochrome C reductase core (1:4 000; Abcam, ab110252), anti-ubiquinone (1:500; Proteintech, 17812-1-AP), and anti-FSP1 (1:500; Proteintech, 20886-1-AP). Thereafter, the membranes were incubated with goat anti-mouse/anti-rabbit secondary antibodies (1:5 000; Proteintech, SA00001-1, SA00001-2) conjugated with horseradish peroxidase (HRP) at RT for 2 h, followed by detection using an enhanced chemiluminescence detection kit (P10100, NCM Biotech), and images were captured using a chemiluminescence imaging system (Tanon 5800, Tanon Science & Technology Co., Ltd. Shanghai, China). β-actin (1:20 000; Proteintech, 66009-1-Ig) was used as an internal control.

### Multiplex Immunohistochemistry (mIHC)

The expression and localization of proteins involved in apoptosis and ferroptosis were identified using mIHC modified for adherent cells (patent pending), using a PANO Multiplex IHC Kit (0001100100; Panovue, Beijing, China).

### Statistical Analysis

Quantitative variables were expressed as the mean ± standard deviation, and Student’s *t*-test was used to analyze the differences between the two groups. *P* < 0.05 was considered statistically significant. All data were analyzed using GraphPad Prism 8.2.0 (GraphPad Software Inc., San Diego, CA, USA).

## Results

### Propofol Inhibited Proliferation, and Propofol/PIE/Fospropofol Disodium Enhanced Sensitivity to Doxorubicin and Paclitaxel of MDA-MB-231 Cells

**Figure 1:**
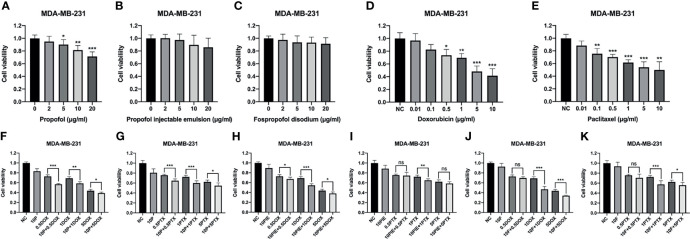
Cell viability of MDA-MB-231 cells treated with propofol/PIE/fospropofol disodium, doxorubicin/paclitaxel, or drug combinations at different concentrations. **(A–C)** Effects of propofol, PIE or fospropofol disodium on MDA-MB-231 cell viability. **(D, E)** Effects of doxorubicin or paclitaxel on MDA-MB-231 cell viability. **(F, G)** Effects of propofol with doxorubicin or paclitaxel on MDA-MB-231 cell viability. **(H, I)** Effects of PIE with doxorubicin or paclitaxel on MDA-MB-231 cell viability. **(J, K)** Effects of fospropofol disodium with doxorubicin or paclitaxel on MDA-MB-231 cell viability. Cell viability was determined using CCK-8 assays, and each experiment was repeated at least three times. NC, normal control; 10P, 10 µg/ml propofol; 10PIE, 10 µg/ml propofol injectable emulsion; 10F, 10 µg/ml fospropofol disodium; 0.5DOX, 0.5 µg/ml doxorubicin; 1DOX, 1 µg/ml doxorubicin; 5DOX, 5 µg/ml doxorubicin; 0.5PTX, 0.5 µg/ml paclitaxel; 1PTX, 1 µg/ml paclitaxel; 5PTX, 5 µg/ml paclitaxel. ns, no significant difference. *P < 0.05, **P < 0.01, ***P < 0.001.

The viability of MDA-MB-231 cells was determined using a CCK-8 assay after treatment with 0-20 µg/ml propofol, PIE, or fospropofol disodium, or 0.01-10 µg/ml doxorubicin or paclitaxel for 24 h. As shown in **Figure 1**, exposure to propofol resulted in a significant dose-dependent decrease in cell proliferation compared with control (**Figure 1A**), while proliferation was inhibited following treatment with PIE or fospropofol disodium, although not significantly (**Figures 1B, C**). Cell proliferation was inhibited in a dose-dependent manner by either doxorubicin or paclitaxel (**Figures 1D, E**).

To investigate whether propofol/PIE/fospropofol disodium has synergistic effects with doxorubicin or paclitaxel, we administered 10 µg/ml propofol/PIE/fospropofol disodium together with 0.5, 1, and 5 µg/ml doxorubicin or paclitaxel to MDA-MB-231 cells. As shown in **Figures 1F–K**, combination with propofol induced significant inhibition of proliferation compared with that of doxorubicin alone (cell viability, 72.83 ± 3.22% vs. 56.90 ± 1.42%, *P* < 0.001; 69.09 ± 3.24% vs. 58.64 ± 3.38%, *P* < 0.01; 43.75 ± 2.92% vs. 38.93 ± 2.22%, *P* < 0.05, respectively). Similarly, propofol combined with paclitaxel significantly inhibited cell proliferation compared with that of paclitaxel alone (cell viability, 75.73 ± 2.15% vs. 64.68 ± 3.76%, *P* < 0.001; 72.21 ± 3.35% vs. 59.94 ± 4.43%, *P* < 0.001; 62.29 ± 3.67% vs. 54.61 ± 5.60%, *P* < 0.05, respectively). Consistently, similar results were obtained from PIE and fospropofol disodium when combined with doxorubicin or paclitaxel. Based on the results above, we selected 10 µg/ml propofol/PIE/fospropofol disodium together with 1 µg/ml doxorubicin or paclitaxel for further experiments.

### Propofol/PIE/Fospropofol Disodium Induced MDA-MB-231 Cell Apoptosis

**Figure 2:**
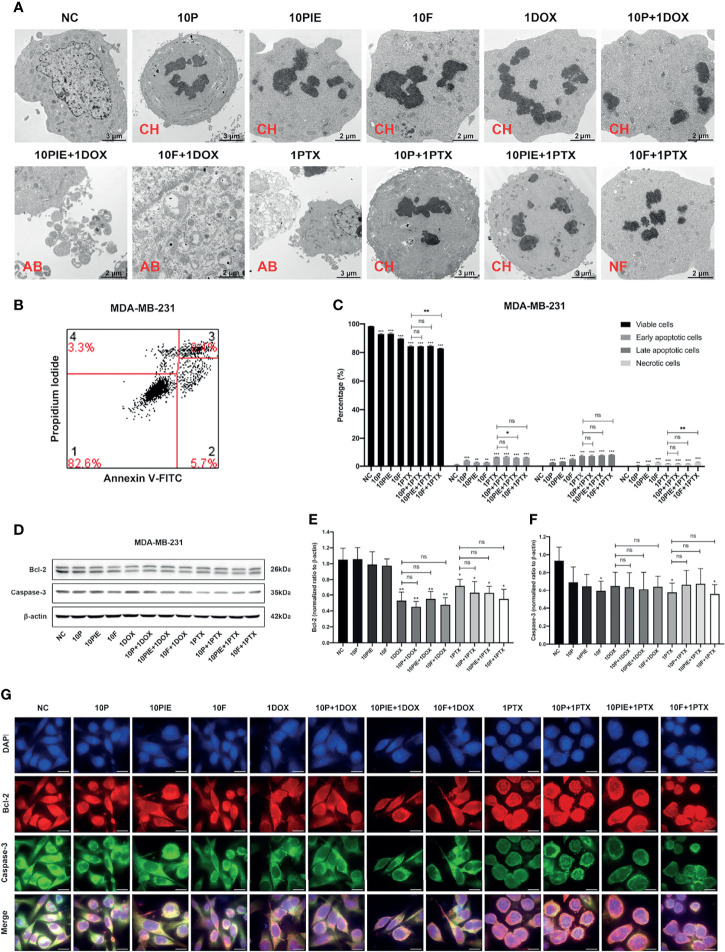
Apoptosis analyses of MDA-MB-231 cells exposed to propofol/PIE/fospropofol disodium with or without doxorubicin/paclitaxel. **(A)** Apoptotic morphological changes of MDA-MB-231 cells treated with different drugs, observed *via* TEM. CH, chromatin condensation; AB, apoptotic bodies; NF, nucleus fragmentation. **(B)** Gating strategy for cell apoptosis assessment. Representative plots were obtained from MDA-MB-231 cells administered fospropofol disodium (10 µg/ml) plus paclitaxel (1 µg/ml). **(C)** Percentage of viable, early and late apoptotic, and necrotic MDA-MB-231 cells exposed to propofol/PIE/fospropofol disodium with or without paclitaxel. **(D)** The expression level of Bcl-2 and caspase-3 in MDA-MB-231 cells under treatment with different drugs was determined by western blotting. β-actin was used as the internal reference. **(E, F)** The statistical analyses of western blotting bands of Bcl-2 and caspase-3. Each experiment was repeated at least three times. **(G)** The expression and subcellular localization of Bcl-2 and caspase-3 in MDA-MB-231 cells after different drug treatments was determined by mIHC. DAPI was used to stain cell nuclei. Bar = 14 µm. NC, normal control; 10P, 10 µg/ml propofol; 10PIE, 10 µg/ml propofol injectable emulsion; 10F, 10 µg/ml fospropofol disodium; 1DOX, 1 µg/ml doxorubicin; 10P+1DOX, 10 µg/ml propofol combined with 1 µg/ml doxorubicin; 10PIE+1DOX, 10 µg/ml PIE combined with 1 µg/ml doxorubicin; 10F+1DOX, 10 µg/ml fospropofol disodium combined with 1 µg/ml doxorubicin; 1PTX, 1 µg/ml paclitaxel; 10P+1PTX, 10 µg/ml propofol combined with 1 µg/ml paclitaxel; 10PIE+1PTX, 10 µg/ml PIE combined with 1 µg/ml paclitaxel; 10F+1PTX, 10 µg/ml fospropofol disodium combined with 1 µg/ml paclitaxel. ns, no significant difference. *P < 0.05, **P < 0.01, ***P < 0.001.

We analyzed MDA-MB-231 cell apoptosis following different drug treatments. Apoptotic morphological changes, such as chromatin condensation, apoptotic bodies, and nuclear fragmentation, were observed using TEM in all drug-treated groups (**Figure 2A**).

Flow cytometry was used to determine the percentage of apoptotic cells (**Figures 2B, C**). Considering the spontaneous fluorescence of doxorubicin, this analysis was not performed in the doxorubicin-treated groups. As can be seen from **Figure 2C**, compared with the control group, cells exposed to 10 µg/ml propofol/PIE/fospropofol disodium alone were associated with a significantly higher percentage of early apoptotic cells (3.88 ± 0.39% vs. 1.10 ± 0.20%, *P* < 0.001; 2.61 ± 0.29% vs. 1.10 ± 0.20%, *P* < 0.01; 2.57 ± 0.26% vs. 1.10 ± 0.20%, *P* < 0.01, respectively), late apoptotic cells (2.46 ± 0.27% vs. 0.31 ± 0.03%, *P* < 0.001; 3.17 ± 0.09% vs. 0.31 ± 0.03%, *P* < 0.001; 4.92 ± 0.43% vs. 0.31 ± 0.03%, *P* < 0.001, respectively), and necrotic cells (0.72 ± 0.13% vs. 0.17 ± 0.04%, *P* < 0.01; 1.04 ± 0.13% vs. 0.17 ± 0.04%, *P* < 0.001; 2.74 ± 0.27% vs. 0.17 ± 0.04%, *P* < 0.001, respectively). The percentage of early apoptotic cells significantly decreased after treatment with 10 µg/ml PIE plus 1 µg/ml paclitaxel, compared with that of paclitaxel treatment alone (5.75 ± 0.20% vs. 6.30 ± 0.13%, *P* < 0.05), while the other two combination treatment groups did not significantly differ from paclitaxel treatment alone. No significant difference was observed in late apoptotic cells between the combination treatment groups and the paclitaxel alone group. In addition, although more necrotic cells were found in the 10 µg/ml fospropofol disodium plus 1 µg/ml paclitaxel treatment than with paclitaxel only (2.95 ± 0.28% vs. 1.97 ± 0.17%, *P* < 0.01), the other two combination treatment groups did not significantly differ with the paclitaxel alone treatment.

The expression and localization of apoptosis-related proteins were determined using western blotting and mIHC. As shown in **Figures 2D–F**, the expression levels of Bcl-2 and caspase-3 decreased in the groups treated with different drugs compared with control group. Both Bcl-2 and caspase-3 were mainly expressed in the cytoplasm (**Figure 2G**).

### Propofol or PIE Increased Intracellular ROS Level of MDA-MB-231 Cells With or Without Doxorubicin or Paclitaxel

**Figure 3:**
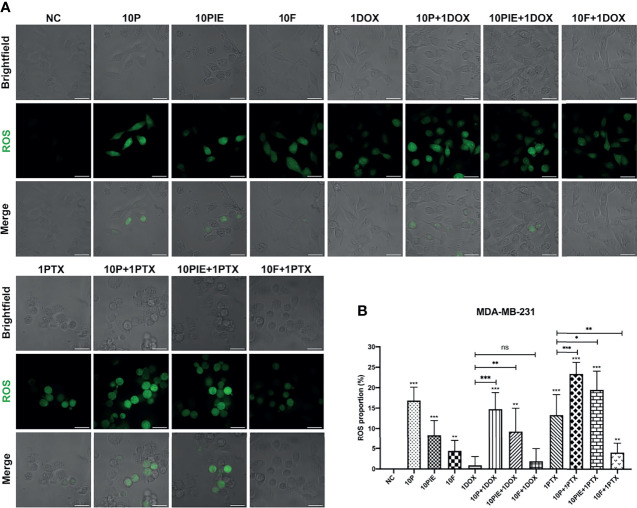
Intracellular ROS level of MDA-MB-231 cells treated with propofol/PIE/fospropofol disodium with or without doxorubicin/paclitaxel. **(A)** Intracellular ROS level of MDA-MB-231 cells treated with different drugs. ROS positive cells were determined using laser scanning confocal microscopy and indicated as green spots. Bar = 34 µm. **(B)** Statistical analysis of ROS proportion in MDA-MB-231 cells under different treatments. ns, no significant difference. *P < 0.05, **P < 0.01, ***P < 0.001.

Intracellular ROS plays a critical role in cell death, it can not only activate the apoptotic signaling pathways, but also induce cell ferroptosis (26). ROS levels in MDA-MB-231 cells were analyzed using fluorescent assay and presented as the proportion of cells marked by green fluorescence (ROS-positive cells) in each merged field. As shown in **Figure 3A**, ROS-positive cells increased after treatment with propofol/PIE (10 µg/ml), 1 µg/ml paclitaxel, or a combination of propofol/PIE (10 µg/ml) and doxorubicin/paclitaxel (1 µg/ml) for 24 h. Intracellular ROS level was significantly higher in MDA-MB-231 cells administered propofol/PIE/fospropofol disodium (10 µg/ml) alone than those in the control (ROS proportion, 16.82 ± 3.31% vs. 0.00 ± 0.00%, *P* < 0.001; 8.36 ± 3.59% vs. 0.00 ± 0.00%, *P* < 0.001; 4.40 ± 2.56% vs. 0.00 ± 0.00%, *P* < 0.01, respectively; **Figure 3B**). As for the combination drug treatments, there was a significant increase in intracellular ROS level in cells treated with propofol/PIE (10 µg/ml) plus doxorubicin (1 µg/ml) compared with doxorubicin alone (ROS proportion, 14.74 ± 4.05% vs. 0.88 ± 2.15%, *P* < 0.001; 9.26 ± 5.71% vs. 0.88 ± 2.15%, *P* < 0.01, respectively; **Figure 3B**), and so did propofol/PIE (10 µg/ml) plus paclitaxel (1 µg/ml) versus paclitaxel alone (ROS proportion, 23.36 ± 2.84% vs. 13.29 ± 5.02%, *P* < 0.001; 19.45 ± 4.61% vs. 13.29 ± 5.02%, *P* < 0.05, respectively; **Figure 3B**).

Interestingly, the effects of fospropofol disodium on intracellular ROS levels relatively differed from those of propofol and PIE. Although fospropofol disodium alone was associated with higher ROS levels in MDA-MB-231 cells relative to the control (4.40 ± 2.56% vs. 0.00 ± 0.00%, *P* < 0.01), it showed no significant difference between fospropofol disodium plus doxorubicin and doxorubicin alone (1.85 ± 3.10% vs. 0.88 ± 2.15%, *P* = 0.541, **Figure 3B**). Moreover, ROS-positive cells were fewer after treatment with fospropofol disodium plus paclitaxel than paclitaxel alone (4.05 ± 2.33% vs. 13.29 ± 5.02%, *P* < 0.001, **Figure 3B**).

### Propofol/PIE/Fospropofol Disodium With or Without Doxorubicin/Paclitaxel Induced Ferroptosis-Related Morphological Changes in MDA-MB-231 Cells

**Figure 4:**
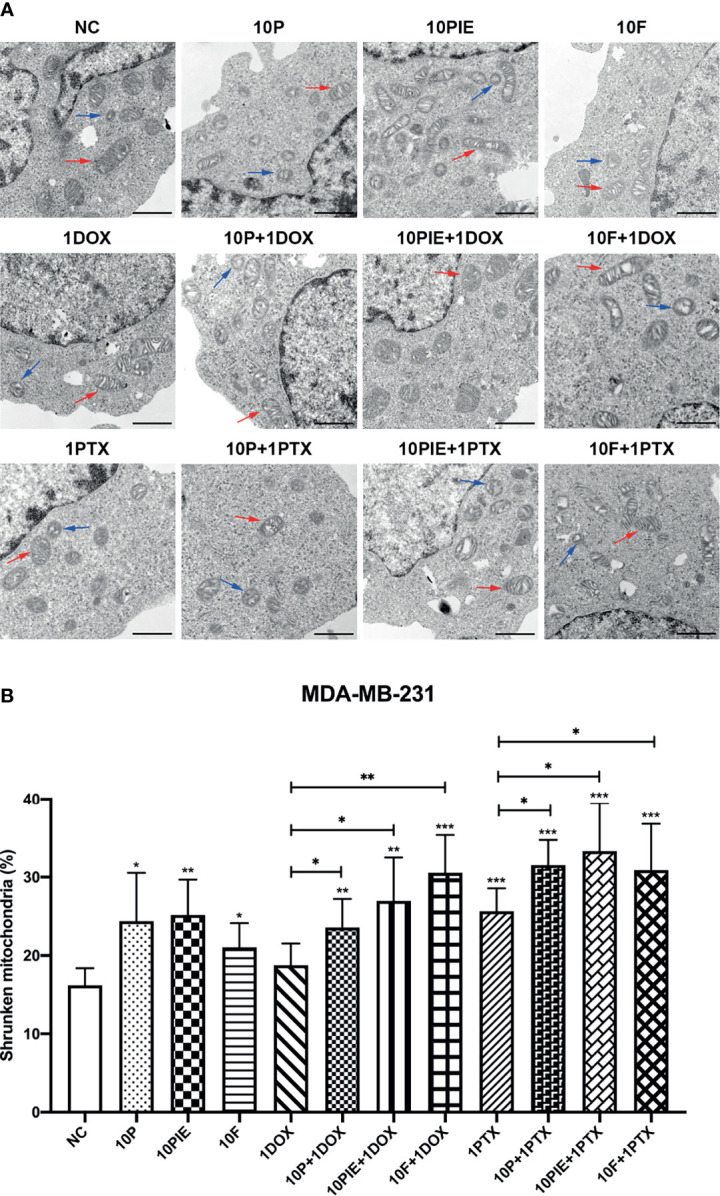
Ferroptosis-related mitochondria morphological changes in MDA-MB-231 cells administered propofol/PIE/fospropofol disodium with or without doxorubicin/paclitaxel. **(A)** Ferroptosis-related mitochondria morphological changes in MDA-MB-231 cells treated with different drugs were determined using TEM. Red arrows: normal mitochondria. Blue arrows: shrunken mitochondria. Bar = 1 µm. **(B)** Statistical analysis of shrunken mitochondria percentage in MDA-MB-231 cells under different treatments. *P < 0.05, **P < 0.01, ***P < 0.001.

Shrunken mitochondria, which appeared smaller than normal, with increased membrane density or decreased mitochondrial cristae, have been reported as the distinctive morphological features of ferroptotic cells (18). In the present study, we distinguished these morphological changes using TEM to determine the effects of propofol/PIE/fospropofol disodium with or without chemotherapeutics on MDA-MB-231 cells (**Figure 4A**). As shown in **Figure 4B**, shrunken mitochondria were significantly increased in MDA-MB-231 cells treated with propofol/PIE/fospropofol disodium (10 µg/ml) alone, compared with control (shrunken mitochondria percentage, 24.39 ± 6.17% vs. 16.22 ± 2.17%, *P* < 0.05; 25.18 ± 4.50% vs. 16.22 ± 2.17%, *P*<0.01; 21.06 ± 3.08% vs. 16.22 ± 2.17%, *P* < 0.05, respectively). Similarly, 1 µg/ml paclitaxel was also associated with a significant increase in shrunken mitochondria compared with that in the control (shrunken mitochondria percentage, 25.65 ± 2.91% vs. 16.22 ± 2.17%, *P* < 0.001). When propofol/PIE/fospropofol disodium (10 µg/ml) was combined with doxorubicin (1 µg/ml), there were more shrunken mitochondria in MDA-MB-231 cells than doxorubicin (1 µg/ml) only (shrunken mitochondria percentage, 23.57 ± 3.64% vs. 18.78 ± 2.76%, *P* < 0.05; 26.98 ± 5.52% vs. 18.78 ± 2.76%, *P* < 0.05; 30.55 ± 4.81% vs. 18.78 ± 2.76%, *P* < 0.01, respectively). Similar results were obtained from propofol/PIE/fospropofol disodium (10 µg/ml) plus paclitaxel (1 µg/ml) (shrunken mitochondria percentage, 31.54 ± 3.20% vs. 25.65 ± 2.91%, *P* < 0.05; 33.32 ± 6.12% vs. 25.65 ± 2.91%, *P* < 0.05; 30.88 ± 5.93% vs. 25.65 ± 2.91%, *P* < 0.05, respectively).

### Propofol/PIE/Fospropofol Disodium With or Without Doxorubicin/Paclitaxel Induced Intracellular Iron Accumulation in MDA-MB-231 Cells

**Figure 5:**
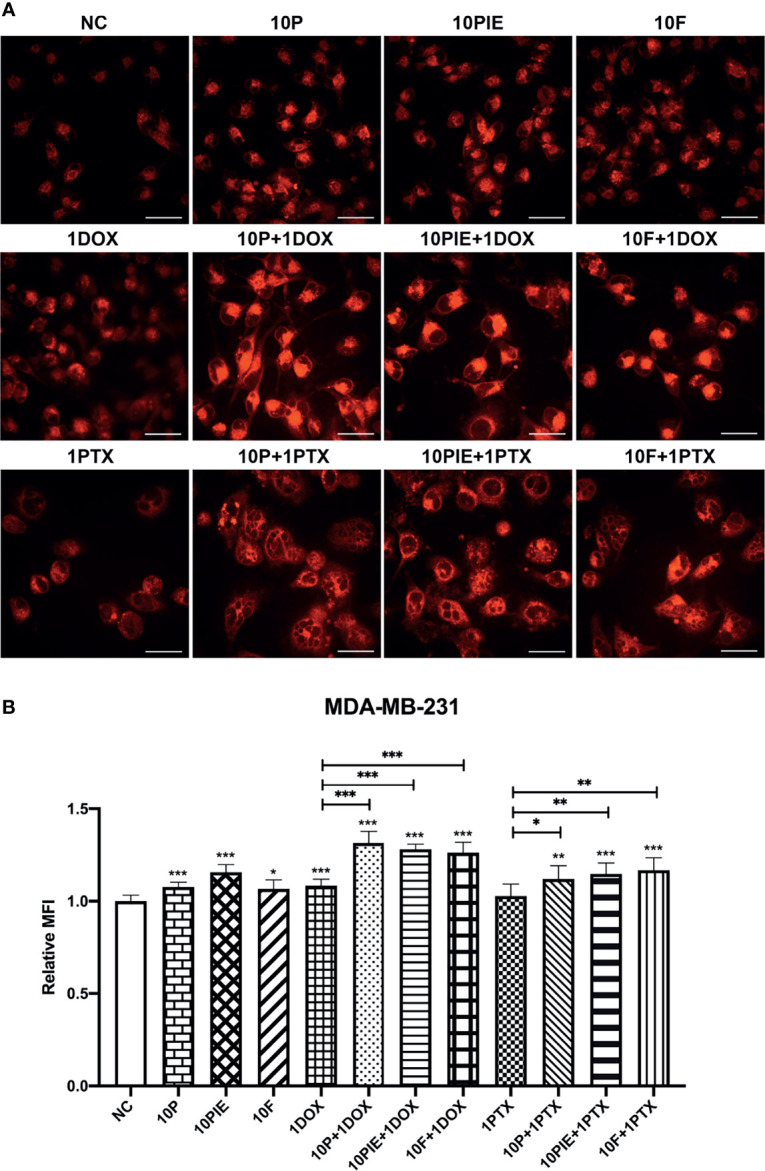
Intracellular iron levels of MDA-MB-231 cells treated with propofol/PIE/fospropofol disodium with or without doxorubicin/paclitaxel. **(A)** Intracellular ferrous ion levels in MDA-MB-231 cells treated with different drugs were determined using laser scanning confocal microscopy. Bar = 34 µm. **(B)** Statistical analysis of relative MFI of intracellular ferrous ions in MDA-MB-231 cells under different treatments. *P < 0.05, **P < 0.01, ***P < 0.001.

Iron overload is also one of the most important characteristics of cell ferroptosis (18). To assess intracellular iron levels, we determined the MFI of Fe^2+^ in MDA-MB-231 cells using laser scanning confocal microscopy. The fluorescent images of intracellular Fe^2+^ are shown in **Figure 5A**. Statistical analyses indicated that cells exposed to propofol/PIE/fospropofol disodium (10 µg/ml) alone were associated with a higher intracellular iron level than those in the control (relative MFI, 1.08 ± 0.03 vs. 1.00 ± 0.03, *P* < 0.001; 1.16 ± 0.04 vs. 1.00 ± 0.03, *P* < 0.001; 1.07 ± 0.05 vs. 1.00 ± 0.03, *P* < 0.05, respectively; **Figure 5B**). Following treatment with 1 µg/ml doxorubicin alone, ferrous ions accumulated more in MDA-MB-231 cells than in the control (relative MFI, 1.08 ± 0.03) vs. 1.00 ± 0.03, *P* < 0.001, **Figure 5B**). Compared with doxorubicin (1 µg/ml) alone, the relative MFI of Fe^2+^ significantly increased after exposure to a combination of propofol/PIE/fospropofol disodium (10 µg/ml) and doxorubicin (1 µg/ml) (relative MFI, 1.32 ± 0.06 vs. 1.08 ± 0.03, *P* < 0.001; 1.28 ± 0.03 vs. 1.08 ± 0.03, *P* < 0.001; 1.26 ± 0.06 vs. 1.08 ± 0.03, *P* < 0.001, respectively; **Figure 5B**), similar to the results of the paclitaxel groups (relative MFI, 1.12 ± 0.07 vs. 1.03 ± 0.06, *P* < 0.05; 1.15 ± 0.06 vs. 1.03 ± 0.06, *P* < 0.01; 1.17 ± 0.07 vs. 1.03 ± 0.06, *P* < 0.01, respectively; **Figure 5B**).

### Propofol May Promote Ferroptosis of MDA-MB-231 Cells *via* the p53-SLC7A11-GPX4 Pathway

**Figure 6:**
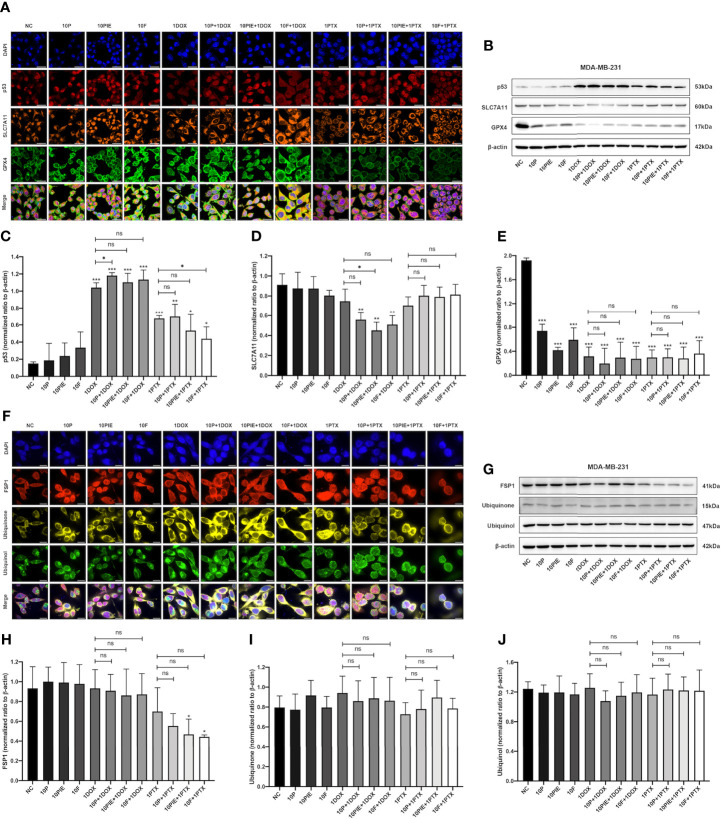
Effects of propofol/PIE/fospropofol disodium with or without doxorubicin/paclitaxel on p53-SLC7A11-GPX4 and ubiquinone-FSP1-ubiquinol signaling pathways in MDA-MB-231 cells. The expression and subcellular localization of p53/SLC7A11/GPX4 **(A)** and FSP1/ubiquinone/ubiquinol **(F)** were determined using mIHC. DAPI was used to stain cell nuclei. Bar = 50 µm **(A)** and 14 µm **(F)**. The expression of p53/SLC7A11/GPX4 **(B)** and FSP1/ubiquinone/ubiquinol **(G)** were determined by western blotting. β-actin was used as the internal reference. The corresponding statistical analyses of western blotting bands of p53 **(C)**, SLC7A11 **(D)**, GPX4 **(E)**, FSP1 **(H)**, ubiquinone **(I)** and ubiquinol **(J)** were conducted and each experiment was repeated at least three times. ns, no significant difference. *P < 0.05, **P < 0.01, ***P < 0.001.

**Figure 7:**
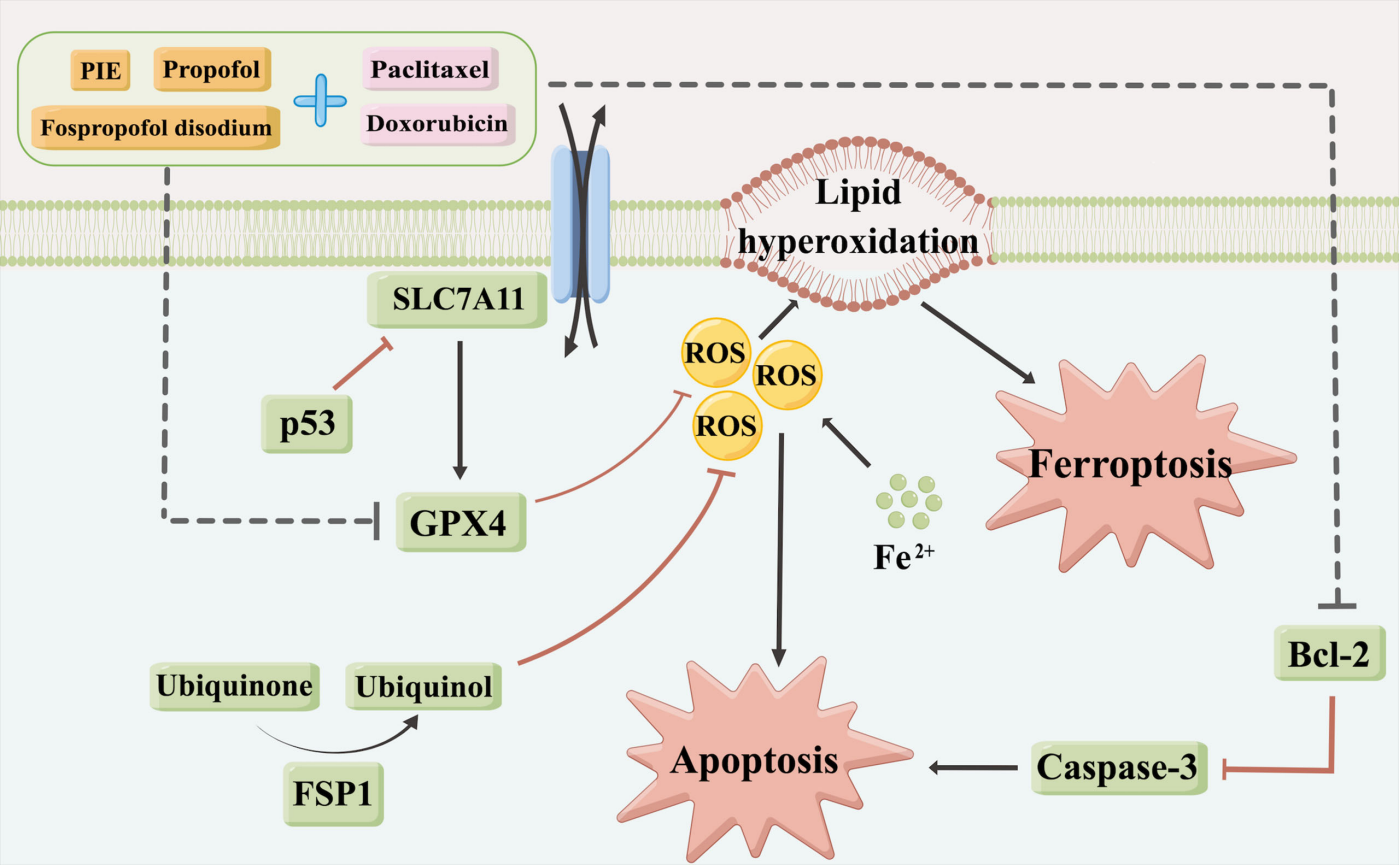
A schematic diagram of potential mechanisms for drug treatment caused apoptosis and ferroptosis in this study (By Figdraw, www.figdraw.com).

The SLC7A11-GPX4 axis and ubiquinone-FSP1-ubiquinol axis were examined to explore the potential mechanisms of propofol-induced ferroptosis-related changes in MDA-MB-231 cells. The locations of p53, SLC7A11, GPX4, ubiquinone, FSP1 and ubiquinol in MDA-MB-231 cells were determined using mIHC and are shown in **Figures 6A, F**, from where it can be observed that p53 is localized in the cytoblast, SLC7A11 mainly on the membrane, and GPX4, ubiquinone, FSP1, and ubiquinol in both mitochondria and cytoplasm. Western blotting was used for quantitative analyses. As shown in **Figures 6B–E**, compared with the control group, after exposure to propofol/PIE/fospropofol disodium (10 µg/ml) alone, the expression levels of GPX4 were significantly decreased. In both doxorubicin- and paclitaxel-related groups, p53 was apparently upregulated, and GPX4 was downregulated compared with the control and other singe propofol-treated groups. SLC7A11 was downregulated in the propofol/PIE/fospropofol disodium plus doxorubicin treatment group compared with doxorubicin alone group. However, although the expression level of FSP1 in cells treated with PIE/fospropofol disodium (10 µg/ml) plus paclitaxel (1 µg/ml) was lower than other groups, there was no significant difference in the expression of ubiquinone and ubiquinol among all groups (**Figures 6G-J**). These results demonstrated that propofol/PIE/fospropofol might promote ferroptosis through regulating the activation of p53-SLC7A11-GPX4 pathway. The schematic diagram of potential mechanisms in this study is shown in **Figure 7**.

## Discussion

Propofol has been commonly used in clinical practice for decades. It was first introduced into clinical treatment in 1986 and soon became one of the most extensively used intravenous general anesthetics to produce sedative and anesthetic effects during surgery (4). At present, surgery is the primary treatment for most tumors, which may cause a large number of cancer cells to be released and reduce the activity of T, B and NK lymphocytes, leading to tumor progression however (27). As a general anesthetic, propofol may play an essential role in tumor relapse and metastasis during surgery process (28). Recent investigations have reported that propofol showed tumor inhibitory effect through promoting cell apoptosis *via* regulating multiple signaling pathways, downstream molecules, microRNAs, and long non-coding RNAs in various tumors, including breast cancer (6–11).

Consistent with previous research, results in this study suggested that propofol could inhibit the proliferation of MDA-MB-231 cells and increase their sensitivity to doxorubicin and paclitaxel. Further apoptotic analyses indicated that these effects of propofol may be mediated through inducing cell apoptosis, with obvious apoptotic morphological changes and higher apoptotic cell proportions in drug treatment groups. Moreover, the expression levels of Bcl-2 and caspase-3 also decreased. Next, intracellular ROS levels of MDA-MB-231 cells were examined and found to be significantly higher in the propofol and PIE treatment groups, with or without chemotherapeutics. Intracellular ROS is closely related to cell death and plays a critical role in both apoptosis and ferroptosis. It could not only stimulate the activation of both intrinsic and extrinsic apoptotic signaling pathways, but also induce cell ferroptosis (26). Accordingly, we speculated that, besides inducing cell apoptosis, propofol might inhibit the proliferation of MDA-MB-231 cells and enhance the anti-tumor effects of doxorubicin and paclitaxel also by promoting ferroptosis.

Ferroptosis, a novel form of regulated cell death characterized by mitochondrial shrinkage and the accumulation of intracellular iron and lipid ROS (18, 29), has been found to play an increasingly important role in an increasing number of diseases since its discovery and definition in 2012 (20, 21). Inducing ferroptosis may be an effective tumor treatment strategy (19). In our study, in addition to ROS levels, we also examined mitochondrial morphological changes, intracellular iron levels, and protein expression in two key ferroptosis regulation pathways to find the evidence of ferroptosis in MDA-MB-231 cells. Our results revealed that propofol induced ferroptosis-related mitochondrial morphological changes and enhanced intracellular iron accumulation in MDA-MB-231 cells, which indicated propofol might augment MDA-MB-231 cell ferroptosis, thereby inhibiting cell proliferation. Moreover, this effect may be achieved by regulating the activation of p53-SLC7A11-GPX4 pathway.

The SLC7A11-GPX4 axis is the core signaling pathway in lipid peroxidation and ferroptosis (23). GPX4, glutathione peroxidase 4, uses glutathione to eliminate phospholipid peroxides and prevent ferroptosis. The glutathione is generated from intracellular cysteine, which can be obtained from extracellular cystine through a cystine-glutamate reverse transporter, system x_c_^-^. SLC7A11 is one of the two subunits comprising system x_c_^-^ and can be inhibited by the tumor suppressor p53 (30). Once SLC7A11 is inhibited, system x_c_^-^ will be inhibited and reduce the exchange of intracellular glutamate and extracellular cystine, then the synthesis of glutathione, the substrate of GPX4, will be impeded, inhibiting the GPX4 function and leading to the ROS accumulation and the further ferroptosis. As shown in our study, GPX4 was significantly downregulated in all drug-treated MDA-MB-231 cells, with p53 apparently upregulated in chemotherapy-related groups and SLC7A11 obviously downregulated only in the propofol/PIE/fospropofol disodium plus doxorubicin treatment group. Only the expressions of GPX4 were significantly decreased in single propofol-treated groups, while no significant changes were observed in p53 and SLC7A11. From these results we speculated that propofol was more likely to promote ferroptosis by downregulating GPX4. The further mechanisms by which propofol downregulates GPX4 needs to be further elucidated.

The ubiquinone-FSP1-ubiquinol axis is another essential pathway regulating cell ferroptosis independent of the SLC7A11-GPX4 axis (24, 25). FSP1 regenerates ubiquinol from ubiquinone, and suppresses lipid peroxidation and ferroptosis. Western blotting results in our study showed no significant difference in the expression of ubiquinone and ubiquinol among all treatment groups, but FSP1 was evidently downregulated in paclitaxel-treated groups, and its expression was even lower in the propofol/PIE/fospropofol disodium plus paclitaxel treatment group compared with that in paclitaxel alone group. This indicated that, although propofol may not regulate ferroptosis through the ubiquinone-FSP1-ubiquinol pathway, it may promote ferroptosis *via* other mechanisms mediated by FSP1, which need to be conducted in the future.

TNBC accounts for 10-15% of all breast cancers and usually appears in the form of high-grade invasive ductal carcinoma (31). The evolution of endocrine therapy and anti-HER2 targeted treatment for other subtypes has significantly improved prognoses in these patients (32, 33), but the clinical outcomes for TNBC remain unsatisfactory, with the median overall survival only approximately 18 months (34). Chemotherapy is the primary adjuvant treatment for patients with TNBC. Considering its poor prognosis after surgery, we intended to explore whether propofol could improve the sensitivity of TNBC cells to chemotherapeutics. In this study, MDA-MB-231 cells, which are TNBC cells, and doxorubicin and paclitaxel, which are chemotherapeutics commonly used for TNBC in clinical practice, were selected for the experiments. Our results suggested that propofol could enhance the inhibitory effects of these two chemotherapeutics against MDA-MB-231 cell proliferation by promoting cell ferroptosis to some extent.

Currently, most *in vitro* studies use propofol only to conduct the investigations. However, PIE is the most common formulation of propofol in clinical practice. Fospropofol disodium is a propofol prodrug with good water solubility, need no lipid emulsion as a drug carrier, metabolized into the active metabolite propofol, and inducing an anesthetic effect (35). In this study, we used these two different propofol formulations and included propofol itself, to investigate whether they are consistent in effects on TNBC cells. Considering the commonly used blood concentration of propofol in clinical practice and the dosage used in previous *in vitro* studies (15, 17), we selected 2, 5, 10 and 20 µg/ml for three different propofol formulations to conduct experiments, and chose the most suitable concentration (10 µg/ml) for combination with chemotherapy according to the results of cell proliferation analyses. As shown in our results, MDA-MB-231 cells exposed to propofol showed a significant dose-dependent decrease in cell proliferation compared with the control, while the inhibition of proliferation when treated with PIE or fospropofol disodium was not significant. When combined with doxorubicin or paclitaxel, all three formulations significantly inhibited cell proliferation compared with doxorubicin or paclitaxel alone. The results of ferroptosis-related morphological changes, intracellular iron levels, and expression of ferroptosis-related proteins were similar among the three propofol formulations. As for the intracellular ROS levels, fospropofol disodium considerably differed from the other two formulations. Although fospropofol disodium alone was associated with higher intracellular ROS levels relative to the control, there was no significant difference between fospropofol disodium plus doxorubicin and doxorubicin alone, and even lower ROS levels in the fospropofol disodium plus paclitaxel group compared with paclitaxel alone. The intracellular ROS levels were similar in the three fospropofol disodium treatment groups. We speculate that PIE and fospropofol disodium may have different anti-proliferation mechanisms because their components are not completely the same as propofol. Furthermore, different water solubility may also lead to different anti-tumor effects. Propofol and PIE are hydrophobic agent, which could be easier taken up into cells through cell membrane and conduct their biological effects, while fospropofol disodium is water-soluble, which may affect its absorption by cells and thus affect its biological effects. Previous research have reported that, the oxidation activity of polyunsaturated fatty acids (PUFA) in membrane phospholipids during cell ferroptosis is competitively influenced by monounsaturated fatty acids (MUFA), indicating that exogenous MUFA may perform ferroptosis resistance (36, 37), which may be another underlying mechanism of different anti-tumor effects between PIE and propofol.

Our study focused on the effects of propofol on breast cancer, and found it could inhibit the proliferation of TNBC cells *in vitro* and enhance their sensitivity to chemotherapeutics. In fact, there are also many studies about the effects of different anesthetics often used in clinical practice on patients under breast cancer surgery. Ecimovic P et al. demonstrated that sevoflurane, one of the most commonly used volatile anesthetics, increased proliferation, migration but not invasion in MDA-MB-231 cells, although observed effect size was small and not dose-dependent (38). Tripolt S et al. found that the common analgesic, opioid, triggered breast cancer metastasis *via* oncogenic JAK1/2-STAT3 signaling to promote epithelial-mesenchymal transition, emphasizing the importance of selective and restricted opioid use (39). Serum from patients receiving propofol/paravertebral anesthesia for breast cancer surgery inhibited proliferation of MDA-MB-231 cells *in vitro*, to a greater extent than that from patients receiving sevoflurane/opioid anesthesia-analgesia (40). In clinical research, results are quite controversial. Some studies showed using propofol during breast cancer surgery could improve the patients’ prognosis compared with using inhalation anesthetics (12), but no significant difference was observed in other studies (13). It is hoped that our results could provide a theoretical support for further clinical research, and assist clinicians in better perioperative management and anesthesia selection for patients with breast cancer.

Our study also had some limitations. We aimed to explore whether propofol could inhibit MDA-MB-231 cell proliferation by affecting cell ferroptosis and analyzed ferroptosis using various methods. Although we also analyzed the expression of two key pathways related to ferroptosis and found a possible regulatory mechanism, we did not test and validate the specific molecules in specific pathways, and did not conducted experiments *in vivo*. Further studies focusing on the specific mechanisms by which propofol regulates cell ferroptosis and including both *in vitro* and *in vivo* experiments should be conducted. On the other hand, as mentioned above, we found the different performances of propofol, PIE and fospropofol disodium in the anti-proliferation effect but did not explore the specific mechanisms between these three drugs. Further research is needed in the future.

## Conclusions

Our study found that propofol showed anti-proliferation effects and could be a potential adjuvant to enhance the chemotherapeutic sensitivity of TNBC cells partly through promoting cell ferroptosis. Future studies are urgently needed to elucidate the potential mechanisms underlying the relationship between propofol and cancer cell ferroptosis.

## Data Availability Statement

The datasets used and/or analyzed in the current study are available from the corresponding authors on reasonable request.

## Author Contributions

CS, PL, LP, and YH designed the study. CS, PL, and MZ performed the experiments and acquired the data under the supervision and guidance of LP. CS performed the statistical analyses and wrote the manuscript. PL and LP revised the manuscript. All authors have read and approved the final version of the manuscript.

## Funding

This study was supported by the Peking Union Medical College Hospital Precipitation and Integration Foundation (ZC201906511). The funder was not involved in the study design, collection, analysis, interpretation of data, the writing of this article or the decision to submit it for publication.

## Conflict of Interest

The authors declare that the research was conducted in the absence of any commercial or financial relationships that could be construed as a potential conflict of interest.

## Publisher’s Note

All claims expressed in this article are solely those of the authors and do not necessarily represent those of their affiliated organizations, or those of the publisher, the editors and the reviewers. Any product that may be evaluated in this article, or claim that may be made by its manufacturer, is not guaranteed or endorsed by the publisher.
